# Potential use of sludge from El Ferrol Bay (Chimbote, Peru) for the production of lipids in the culture of *Scenedesmus acutus* (Meyen, 1829)

**DOI:** 10.1038/s41598-024-52919-2

**Published:** 2024-03-23

**Authors:** Fernando Merino, Sorayda Mendoza, Juan Carhuapoma-Garay, Luis Campoverde-Vigo, Yolanda P. Huamancondor-Paz, Yudith Choque-Quispe, Dianeth Buleje Campos, David Choque-Quispe, Liliana Rodriguez-Cardenas, Guillermo B. Saldaña-Rojas, Rómulo E. Loayza-Aguilar, Gustavo E. Olivos-Ramirez

**Affiliations:** 1 Escuela de Biología en Acuicultura, Universidad Nacional del Santa, Av. Universitaria S/N, Nuevo Chimbote, 02712 Peru; 2 Environmental Engineering Department, Research group for the development of advanced materials for water and food treatment, Advanced Materials Research Laboratory for Water and Food Treatment, Universidad Nacional José María Arguedas, Andahuaylas, 03701 Peru; 3 Agroindustrial Engineering Department, Advanced Materials Research Laboratory for Water and Food Treatment, Universidad Nacional José María Arguedas, Andahuaylas, 03701 Peru; 4 Agroindustrial Engineering Department, Research group for the development of advanced materials for water and food treatment, Advanced Materials Research Laboratory for Water and Food Treatment, Universidad Nacional José María Arguedas, Andahuaylas, 03701 Peru; 5 Advanced Materials Research Laboratory for Water and Food Treatment, Universidad Nacional José María Arguedas, Andahuaylas, 03701 Peru

**Keywords:** Environmental biotechnology, Environmental sciences

## Abstract

Despite the extensive development of microalgae biotechnology, it still requires new methodologies to lower production costs, especially in the field of biofuel production. Therefore, innovative methods that facilitate operations and enable cost-effective production are important in driving this industry. In this study, we propose a new low-cost and easy-to-use procedure, addressed to the generation of a culture medium for *Scenedesmus acutus*. The medium was obtained by thermal reduction of a sludge sample from El Ferrol Bay (Chimbote, Peru), whereby we obtained an aqueous medium. Our results indicated that the aqueous medium incorporates all necessary nutrients for microalgae production; allowing a maximum biomass of 0.75 ± 0.07 g/L with 60% of the medium; while high lipids production (59.42 ± 6.16%) was achieved with 20%. Besides, we quantified, in the experimental medium and at the end of the cultures, the levels of inorganic nutrients such as ammonium, nitrites, nitrates, and phosphates; in addition to COD and TOC, which were significantly reduced ($p<$ 0.05) after 7 days of culture, mainly in the treatment with 20%. These results suggest tremendous potential for sludge reuse, which also entails a cost reduction in microalgae biomass production, with additional positive impacts on large-scale application over highly polluted environments.

## Introduction

Microalgae are a group of photosynthetic microorganisms frequently used for biocompounds production, including fatty acids, pigments, chemicals with antioxidant or anti-inflammatory effects, and lately biohydrogen^1–4^. They afford great advantages aimed at massive production, namely for the fact that they do not require fertile soils for their cultivation, grow in a wide range of environmental conditions, have rapid growth, and can be mass-produced with organic waste components or effluents^5–8^. These advantages have enabled the trade-in microalgae products to reach a value of over US$6.5 billion, out of which about US$2.5 billion is generated by the health food sector, US$1.5 billion by the production of DHA and US$700 million by aquaculture^9^. Despite this, scaling up microalgae cultures encounters major challenges that need to be overcome for sustainable production, especially in the field of biodiesel.

As with most microorganisms, microalgae culture requires the development of a nutrient medium^10^. The ability to achieve this objective relies on the optimization of a complete medium, which must supply all necessary nutrients to obtain the best biomass production with the best bromatological characteristics^11^. Such process is of fundamental importance since the medium integrated with the management of the physical-chemical parameters determines the course of the culture; which may also be advantageous for the production of high quantities of a given molecule^12,13^. In this regard, manufacturers rely on traditional culture mediums, such as Guillard F/2 medium^14^; nevertheless, its use implies high costs for large-scale cultivation. Consequently, many studies emphasize the potential of using waste inputs or effluents as alternative sources of nutrients^15–18^.

Phosphorus and nitrogen are the two principal nutrients in microalgae production. It is well known that productivity is limited by the concentration of these two elements^19,20^ and that one can restrict the metabolic dynamics of the other^21^. Concrete evidence of its effects is that under nitrogen-limited conditions microalgae reduce their growth and increase lipid synthesis^22^. Hence, a microalgal culture medium should primarily consider these two nutrients. It is worth mentioning that waste effluents and organic wastes usually exhibit high quantities of these nutrients^23,24^. This is wherefore they have proven to be effective as an alternative medium after a process of standardization^25–29^. The latter is very likely to be the most important factor since the success of the culture depends on it; upon which, the pursuit by different concentration medium results in a suitable alternative for tracing the optimal concentrations^30^.

An attractive possibility for the development of an alternative medium is the sludge from bays. Sludge bay is generated by the accumulation of organic matter on the seabed, resulting from the discharge of industrial or domestic effluents^31^. In the context of climate change and environmental health, these represent a serious threat to the environment and biodiversity. Moreover, it should be noted that much research has been done to characterize these contaminants^32–34^; however, few strategies have been proposed to mitigate them, and those that have been proposed are often expensive. It is worth highlighting that, comprehensive and openly conducted life cycle assessments (LCA) and techno-economic assessments (TEA) are essential for evaluating the environmental and economic prospects and challenges of a technology or product prior to embarking on industrial implementation^35^. In that sense, the study of alternative mediums for the production of microalgae must be aligned with these principles, in order to obtain the best profitability.

Under this context, the objective of this study was to standardize a methodology that employs sludge from El Ferrol Bay (Chimbote, Peru) as a culture medium for the production of *Scenedesmus acutus* with high lipid contents. The results presented in this paper suggest that our methodology is a feasible option for lipids production from microalgae cultivation. In addition, there are positive environmental implications in the use of sludge for the mass production of *S*. acutus.

## Materials and methods

### Microalga strain

The green microalga *S*. *acutus* (Supplementary Fig. 1A) was provided by Laboratorio de Evaluación de los Recursos Acuáticos y Cultivo de Especies Auxiliares at Universidad Nacional del Santa. This strain is maintained aseptically under laboratory conditions, in 500 mL flasks, with constant aeration at a flow rate of 1 L/min and exposure to a white fluorescent emitting $\approx$12000 lx, at 24:0 photoperiod. Heussler-Merino culture medium (HM medium, Supplementary Table 1) is used due to it enables optimal growth^36^. In order to avoid interference of residues from the HM medium in the inoculum, we did not use the inoculum as mentioned, but rather, we used an inoculum that was previously concentrated and cryopreserved at 4 ^∘^C for one month (Supplementary Fig. 1B).

### Sludge sampling and medium preparation

**Figure 1:**
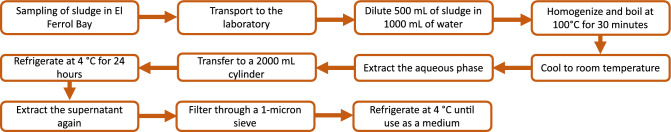
Flow chart of the elaboration of the experimental medium, Aqueous Extract of Sludge (AES). The AES medium was prepared with sludge from El Ferrol Bay, which was obtained by dredging. To withdraw the nutrients, a heat treatment was performed and then the supernatant was extracted. Afterward, the supernatant was filtered and stored at 4 ^∘^C until use.

El Ferrol Bay (Chimbote, Peru) is one of the most polluted ecosystems as a result of decades of dumping of fishing and domestic waste effluents. This has led to the deposit of large volumes of organic matter at the bottom of the bay^37^. For this reason, we collected a sample of 10 kg of sludge by dredging at coordinates 9^∘^04’40”S 78^∘^35’55”W (Supplementary Fig. 2). The sample was immediately transported to the laboratory, where we prepared the culture medium as follows: first, 500 mL of the sludge was diluted in 1000 mL of tap water (1:2 v/v) and the blend was homogenized vigorously in a cooking pot, then, the mixture was heated to 100 ^∘^C and kept under mechanical agitation for 30 minutes and we let it rest until it cools down to room temperature, next, the aqueous phase was carefully transferred to a 2000 mL measuring cylinder, preventing the transfer of coarse particles, after that, we let it stand for 24 hours in refrigeration at 4 ^∘^C to sediment the remaining coarse particles, finally, the supernatant, which we refer to hereafter as aqueous extract of sludge (AES), was extracted, filtered with a 1-micron sieve and refrigerated at 4 ^∘^C until its use as a culture medium (Fig. 1 and Supplementary Fig. 3).

### Nutrient quantification

We quantified 31 elements by Inductively Coupled Plasma (ICP) (Supplementary Table 2), by using iCAP^TM^ 7400 ICP-OES Analyzer. The method is based on the evaporation of the sample, dissociation, ionization, and excitation of the atoms in a plasma. During the process of excitation, electromagnetic radiation emissions are produced in the UV-Vis range, to then be separated according to their wavelength. This process allows determining the presence of an element depending on its emitted wavelength, while its intensity determines the concentration. Sample preparation for this method is relatively simple; it was thermally digested in HNO_3_ (5%), following the recommended standards^38^. Furthermore, we quantified nitrogen in the form of total ammonia nitrogen, by the Macro-Kjeldahl method (4500-Norg B)^39^. Nutrient quantification by ICP was conducted at Laboratorio COLECBI SAC. In addition, chemical oxygen demand (COD) was determined by the EPA 410.4 method^40^, ammonium by EPA 410.4, nitrate by the cadmium reduction method^41^, nitrite by the adapted ferrous sulfate method^42^, and phosphate by the ascorbic acid method^43^. Finally, we performed the analysis of total organic carbon (TOC) by the colorimetric method^44^ with UV detection using commercial kits.

### Cultivation design

Experimental cultures were performed in batch mode for 7 days, under laboratory conditions. We used glass photobioreactors with a culture volume of 500 mL, where we inoculated the *S*. *acutus* strain at an initial concentration of 60 *x* 10^4^ cells/mL. We considered it relevant to evaluate 4 experimental treatments, 20, 40, 60, and 80% of AES (200, 400, 600, and 800 mL/L, respectively), and a control treatment with HM medium. The doses of the experimental treatments were established in view of the level of nitrogen that was quantified in the medium, thus, we have cultures with different nitrogen levels. All treatments were performed in triplicate. The experimental units were maintained with aeration and illumination as mentioned previously.

### Growth measurement

Population growth (cells/mL) was determined daily by microscopic counts with a Neubauer chamber. We followed the suggested recommendations for proper counting^45^. The specific growth rates ($\mu$), divisions per day (*Div*./*day*), and doubling time (*DT*) were calculated using Eqs. (1), (2), and (3), respectively. All equations are described in the literature^46^.

1 $$\begin{aligned} \mu = \frac{ln(N_{2} - N_{1})}{(t_{2} - t_{1})} \end{aligned}$$

2 $$\begin{aligned} Div./day = \frac{\mu }{ln(2)} \end{aligned}$$

3 $$\begin{aligned} DT = \frac{1}{Div./day} \end{aligned}$$

Where $N_1$ and $N_2$ are population density in *x* $10^4$ cells/mL at time 1 ($t_1$) and time 2 ($t_2$), respectively.

### Biomass quantification

The biomass was measured at the end of the experiment by taking a 50 mL aliquot in preweighed Petri dishes. They were oven dried at 55 ^∘^C (overnight). The dry weight of the biomass (g/L) was measured gravimetrically using a ± 0.01 sensitivity balance. To avoid possible bias, we measure the total solids in the medium to subtract from the total weight.

### Population structure

*S*. *acutus* is a microalga that grows forming 1-, 2-, 4- and 8-cell cenobium. Although the factors that determine the number of cenobium are unknown, the number of cenobium can be an indicator of proper growth. Hence, we performed the evaluation of the population structure in all treatments. To this end, we calculated the number of cenobium formed by all cells contained in 25 *x* 10^-5^ mm^3^, similar to another study^47^.

### Lipid content

Total lipid content was determined by a single-step procedure^48^. For that purpose, we took a sample of 15 mL, in pre-weighed test tubes, from each culture to centrifuge at 4000 g for 10 min. Afterward, the liquid phase was removed to obtain the pellet. The pellet was dried in an oven at 55 ^∘^C, overnight. Each sample was weighed to determine the biomass by difference. Subsequently, 3 mL of extraction solution (chloroform and methanol; 2:1 v/v) was added, shaken vigorously, and left for 5 hours. The mixture was centrifuged, the supernatant was decanted, and the pellet was dried again to record the new weight. The lipid content was estimated in percent (%) by the difference of the weights before and after extraction.

### Physical-chemical parameters

pH and temperature are two of the most important parameters in microalgae cultivation. Monitoring their evolution provides important information about the dynamics of the cultures. For this reason, we recorded daily both of them with the aid of a multi-parameter equipment of ± 0.01 sensitivity.

### Parameter correlations

We determined the correlation between the main collected parameters: growth (cells/mL), biomass (g/L), lipid content (%), pH (initial and final), and temperature (^∘^C). For this purpose, we determined Pearson correlation and confidence intervals by bootstrapping the correlated data in R software^49^. Confidence intervals (C.I.) were determined at 95% with 5000 resamples.

### Data analysis

The nutrients quantified by ICP were plotted on a logarithmic scale for better visualization. The population density was plotted as a function of time. Population structure was presented as a percentage in stacked bar charts for 1-, 2-, 4- and 8-cell cenobium. Normality was performed with the Shapiro-Wilk test to determine the normality of the data. To determine significant differences between treatments, we use one-way analysis of variance (ANOVA), with *post hoc* test (Tukey’s method) with a level of 5%, using the Statsmodels module of Python^50^. All plots were performed using the Matplotlib package^51^. The code repository, to reproduce the analysis, can be obtained at the following link: https://github.com/tavolivos/Microalgae-plots.

## Results and discussion

### Medium chemical profile

**Figure 2:**
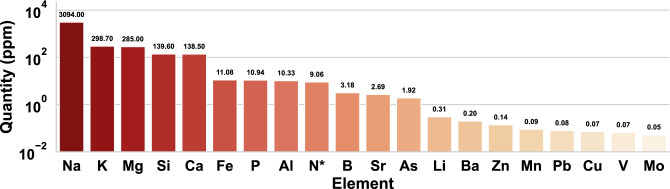
Chemical profile of the AES culture medium. The main elements quantified in the AES medium and their respective individual contributions are presented in ppm. The values are plotted using a logarithmic scale. *Nitrogen was quantified as ammonia nitrogen (NH_3_).

Chemical characterization of the AES medium revealed that it incorporates all the nutrients needed for microalgae cultivation, as well as other highly toxic elements. At least 20 elements were quantified with levels $\ge$ 0.05 ppm. It is crucial to highlight that some of the nutrients with high values occur naturally in seawater, such as sodium, potassium, magnesium, and calcium. Here, we did not consider analyzing aluminum as it is the container material used in the thermal process and there could be detachment during the preparation of the medium. Besides, we identified Mg, Si, Ca, Fe, P, N, B, Sr, As, Li, Ba, and, Zn as the elements with the highest concentration. Other elements with concentrations between 0.09 and 0.05 ppm were Mn, Pb, Cu, V, and Mo; some of which are toxic, like Pb and Cu. Interestingly, the medium showed a balanced ratio of nitrogen and phosphorus, whose values are 9.06 and 10.94 ppm, respectively (Fig. 2).

This study has used a heat treatment approach for the preparation of the culture medium. Thermal treatments are widely used in different technologies, as high temperatures increase the mobility and facilitate the extraction of organic contaminants^52^. In addition, it induces the disruption of cellular components and other complex molecules, such as polymers, proteins, and carbohydrates^53^. The dissociation of polymers to simpler chemical structures is the result of the disruption of chemical bonds, triggered by the high kinetic energy of heating. These low molecular weight compounds, such as sugars, amino acids, or short lipid chains, are more easily incorporated by microalgae and therefore allow for enhanced growth^54,55^. An additional advantage of this methodology is the elimination of microorganisms that may infect microalgae or simply compete for nutrients. Other research has pointed out the possibility of using the waste effluent directly since it contains a greater organic load, as well as microorganisms that produce chemical species that favor the growth of microalgae^56^. According to our criteria, this would produce contamination in the cultures. However, it is possible to use the sludge directly in cases where the objective is only the removal of nutrients and not the production of algal biomass. Therefore, a temperature-based separation methodology to obtain an aqueous medium is the most appropriate.

The culture medium described in this research has been shown to provide elements such as N, P, K, Mg, and Fe, essential for microalgae reproduction^57^. In addition, trace metals, such as Mg, Mn, B, Mo, K, Co, and Zn, are also required as co-factors for cellular functions^58^. All these nutrients, excluding Co, have been quantified in the AES medium with levels > 0.01, whose concentrations are not balanced as in traditional mediums. It is important to note that the nutrient balance could not be controlled since the medium was elaborated from a unique organic component collected from the natural environment. However, having a balanced culture medium is necessary to support a continuous increase in biomass; furthermore, microalgae cultures can be improved only if nutrient deprivation can be controlled throughout cell growth^59^. Therefore, not having a balanced culture medium is a limitation for our procedure, as well as for other studies involving residual material since it is not possible to balance the medium in such cases. Besides, the incorporation of complementary nutrients to balance the medium could be a cost-increasing option. Despite this, the AES medium allowed us to obtain important growth and, in the case of the lowest concentration, high levels of lipids. Further studies could also perform calculations from the unbalanced media to predict biomass yields by theoretical calculations based on the composition of microalgae cells.

It is worth noting that, we identify some elements in the medium that may be limiting the growth due to its toxicity. For instance, As, Cd, Pb, Ba, and Ag. Previous reports indicate that high concentrations of these metals induce oxidative stress, changes in metabolism and structure, and degradation of vesicle cells^60,61^. Although, microalgae possess adaptive mechanisms to bioaccumulate these compounds; among which vacuolization, accumulation in the cell wall, and other processes of resistance induced by metallochaperones^62,63^. Under these circumstances, it is possible to sustain high growth even in the presence of high concentrations of toxic elements.

*Scenedesmus* spp. are well known for their adaptability to different conditions and culture mediums. Toxicity studies suggest that they are able to tolerate a wide range of heavy metals; such as up to 200 ppm Pb^64^, 10 ppm Cr^65^, 100 ppm As^66^, 20 ppm Cd^67^, 5 ppm Ba^68^, and 0.0385 ppm Ag^69^. Growth under these conditions is limited, but possible. *S*. *acutus* is, therefore, one of the most promising microalgae to cultivate with AES medium.

**Table 1:** Chemical analysis of the inorganic nutrients, COD, and TOC of the sludge, AES medium, and water at the end of the experiment.

Sample	COD (mg/L)	Ammonium (mg/L)	Nitrite ($\mu$/L)	Nitrate (mg/L)	Phosphate (mg/L)	TOC (mg/L)
Sludge	22.67 ± 197^a^*	–	–	–	–	0.12 ± 0.002^a^
EAS medium	1766.67 ± 82^b^	42.87 ± 9.33^a^	0.04 ± 0.05^a^	10.67 ± 0.75^a^	22.06 ± 2.32^a^	0.10 ± 0.007^a^
Final culture with 20%	1296.67 ± 31^c^	0.74 ± 0.15^b^	0.20 ± 0.09^b^	12.99 ± 0.19^b^	15.38 ± 1.15^b^	0.06 ± 0.001^b^
Final culture with 40%	1336.67 ± 179^c^	1.27 ± 0.09^c^	0.14 ± 0.06^c^	16.51 ± 1.76^c^	20.93± 3.04^a^	0.12 ± 0.019^a^
Final culture with 60%	1230.00 ± 20^d^	1.55 ± 0.22^c^	0.09 ± 0.06^d^	20.10 ± 7.09^d^	21.20 ± 1.56^a^	0.28 ± 0.023^c^
Final culture with 80%	1353.33 ± 106^c^	6.78 ± 0.84^d^	0.07 ± 0.04^d^	15.59 ± 4.43^c^	40.18 ± 24.35^d^	0.16 ± 0.007^d^

Different letters in the same column indicate significant differences between groups (*p* < 0.05).

*COD in mg/kg.

On the other hand, the analysis of inorganic nutrients demonstrated the reduction of nutrients by the microalga *S*. *acutus*. In relation to COD, the highest level was observed in the EAS medium (1766.67 ± 82 mg/L), while, after 7 days of culture a maximum reduction of 30.37% was achieved in the treatment with 60% EAS medium (1230 ± 20 mg/L). Interestingly, ammonium levels were significantly reduced (p < 0.05), on the last day, with the treatment with 20% AES medium, showing the highest reduction (98.27%). Meanwhile, nitrites remained relatively low (<0.20 $\mu$/L), and nitrates in higher concentration, as normally occurs due to the nitrifying activity of the bacteria^70^. In contrast, phosphates were reduced only in the 20% AES medium treatment by 30.28%, while in the 40 and 60% AES medium treatment, they were reduced by less than 5.1%. Surprisingly, phosphates in the 80% AES medium treatment increased by 82.14%, which we attribute to slow growth and subsequent culture death (Table 1). Likewise, TOC was slightly reduced only in the 20% medium treatment; however, it increased in the other treatments, probably attributed to cell death and the presence of bacteria in the water.

The high concentration of these elements in the AES medium demonstrates dramatic levels of pollution in the sampling area. Additionally, it is essential to note that the concentration of chemical elements present in the sludge may fluctuate; therefore, the final chemical composition of the medium will depend on the characteristics of the sample in the collecting area^71^. Nevertheless, it is expected that the use of our methodology with other samples from nearby places to the collecting area will provide similar results to the obtained data. In this sense, future studies will be required to expand the sampling area and determine the variations in the characteristics of the medium, in function of the geographic location and depth.

### Population growth and biomass production

**Figure 3:**
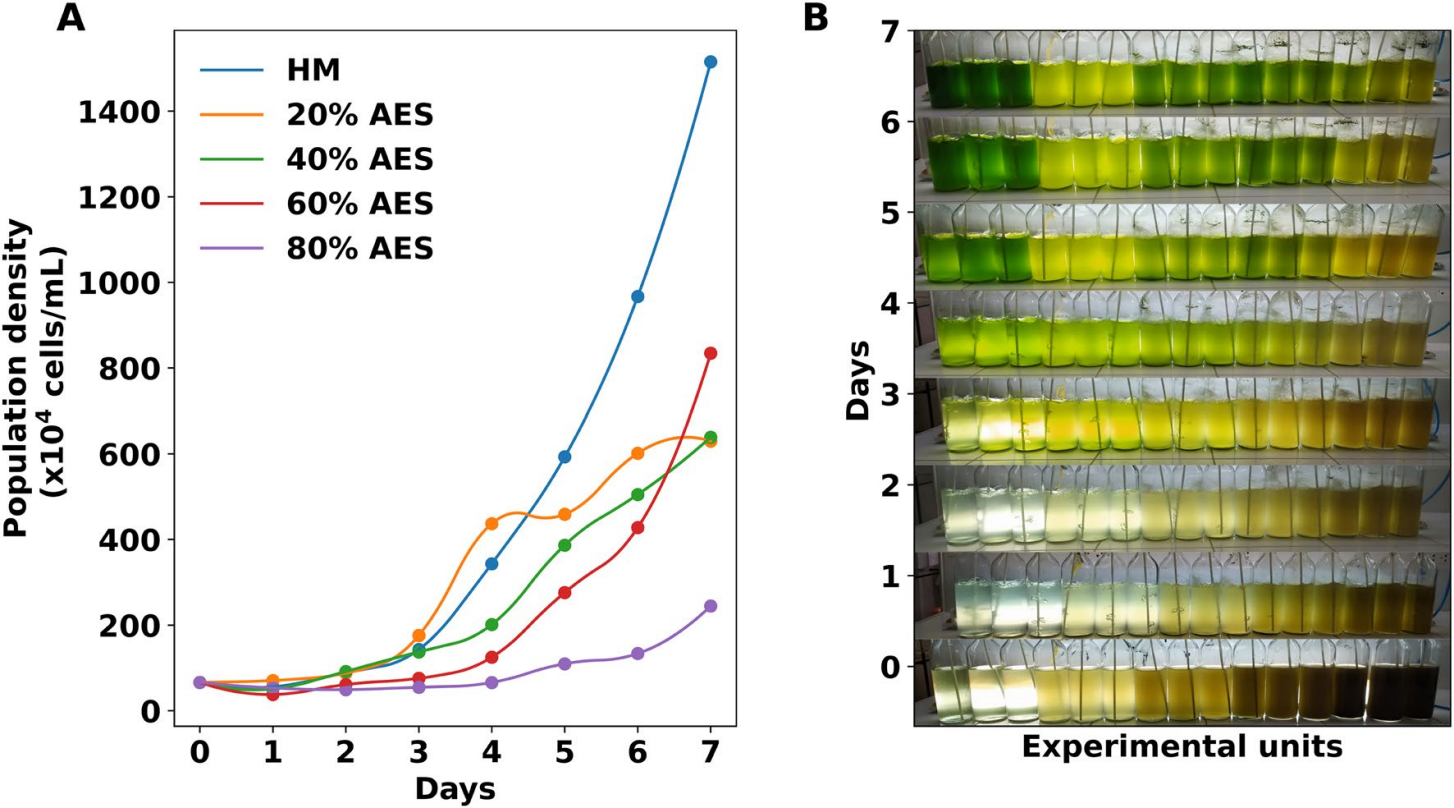
Population growth of the microalgal cultures. (**A**) Growth curves of the average population density. (**B**) Different tonalities of the experimental treatments during the 7 days of cultivation are related to lipids and pigment synthesis depending on nutrient concentration (control treatment, 20, 40, 60, and 80% of AES medium, from left to right, each with 3 replicates).

Our experiments demonstrate that the AES medium allows microalgae growth in all treatments. However, none of them exceeded the population growth obtained with HM medium (Fig. 3A). Maximum growth was obtained with 60% of AES medium (833.61 ± 24.54 *x* 10^4^ cells/mL), which represents almost half of that obtained with HM medium (1515.67 ± 371.98 *x* 10^4^ cells/mL). Interestingly, treatments with 20 and 40% of AES showed similar growth trends (629.00 ± 59.81 and 638.33 ± 28.22 *x* 10^4^ cells/mL), while the lowest growth was achieved with 80% AES (244.00 ± 52.20 *x* 10^4^ cells/mL). It is important to note that treatments showed different tonalities attributed to the synthesis of lipids (yellowish) and pigments (greenish), both of which are associated with nutrient availability, mainly nitrogen (Fig. 3B).

Besides, the statistical analyses of the growth variables were found to correlate with the doses of nutrients supplied (Table 2). We highlight that the specific growth rates ($\mu$) increase with the amount of medium up to 60%. Beyond this concentration, the cultures exhibit a significant decrease in all growth parameters ($\mu$, *Div*/*day*, *DT*, and biomass). Moreover, it is noteworthy that the treatments of 20, 40, and 60% did not present significant statistical differences among them (*p* < 0.05); however, in all cases, the HM medium provides better performance.

Both population density and biomass have been influenced by the AES medium and the conditions that it generates. It has been established that the availability of nitrogen and phosphorus are the main growth-limiting factors^72^; therefore, the increase observed at higher concentrations was expected. Although we have evaluated wide ranges of medium concentrations, we have noted that no experimental concentrations were able to outperform the HM medium.

The cultures supplemented with HM medium presented the best performance, both in population growth and biomass. This medium incorporates 4 nutrients: urea, potassium chloride, phosphoric acid, and iron. The better yields of the control cultures can be attributed to the better balance of this medium, in addition to the fact urea incorporates carbon as a nutrient^73^. In other experimental studies, the biomass of *Scenedesmus* ranges from 0.56^74^ to 1.76 g/L^75^. Those fluctuations depend mainly on physical-chemical factors, as well as on whether the work was performed, indoors or outdoors. Therefore, our results suggest that HM medium leads to 1.69 ± 0.14 g/L, while 60% AES leads to a maximum of 0.75 ± 0.07 g/L, which is about half. Nevertheless, the biomass obtained is considerable enough to justify the use of the AES medium. Likewise, better growth could be obtained by modifying the cultivation parameters or the cultivation strategy.

In regards to growth phases, the lag phase of the cultures was similar in all treatments up to day 2. On the other hand, the 60 and 80% treatments maintained a lag phase up to day 4. This difference between the lag phases could be associated with the toxic elements found, since the 20 and 40% treatments, which contained fewer contaminant concentrations, presented a lower lag phase. This reinforced the excellent adaptability of *S*. *acutus*. In both cases, the period may seem significantly long for production purposes; thus, initial cultures with higher cell density could overcome this disadvantage. Several studies have established that inoculum size influences microalgal cultures^76,77^. In addition, the prolonged lag phase could also be explained by the use of a quiescent inoculum. Considering this, we project that the use of our medium in real scenarios of scaling could lead to higher population growth in less time, and therefore better yields.

On the other hand, the growth variables $\mu$, *Div*./*day*, and *DT*, are in agreement with the nutrient supply. The 80% treatment presented the lowest $\mu$, *Div*./*day*, and the highest *DT*, as a result of the higher amount of contaminants. However, we can also mention that these variables together with the growth could be affected by the turbidity that generates a lower light transmission in the photobioreactor. On the other hand, the 20, 40, and 60% treatments did not show significant differences between these variables. Despite this, we noted other aspects of cellular composition that denoted a change in the metabolism of the microalgae in those.

### Population structure

**Figure 4:**
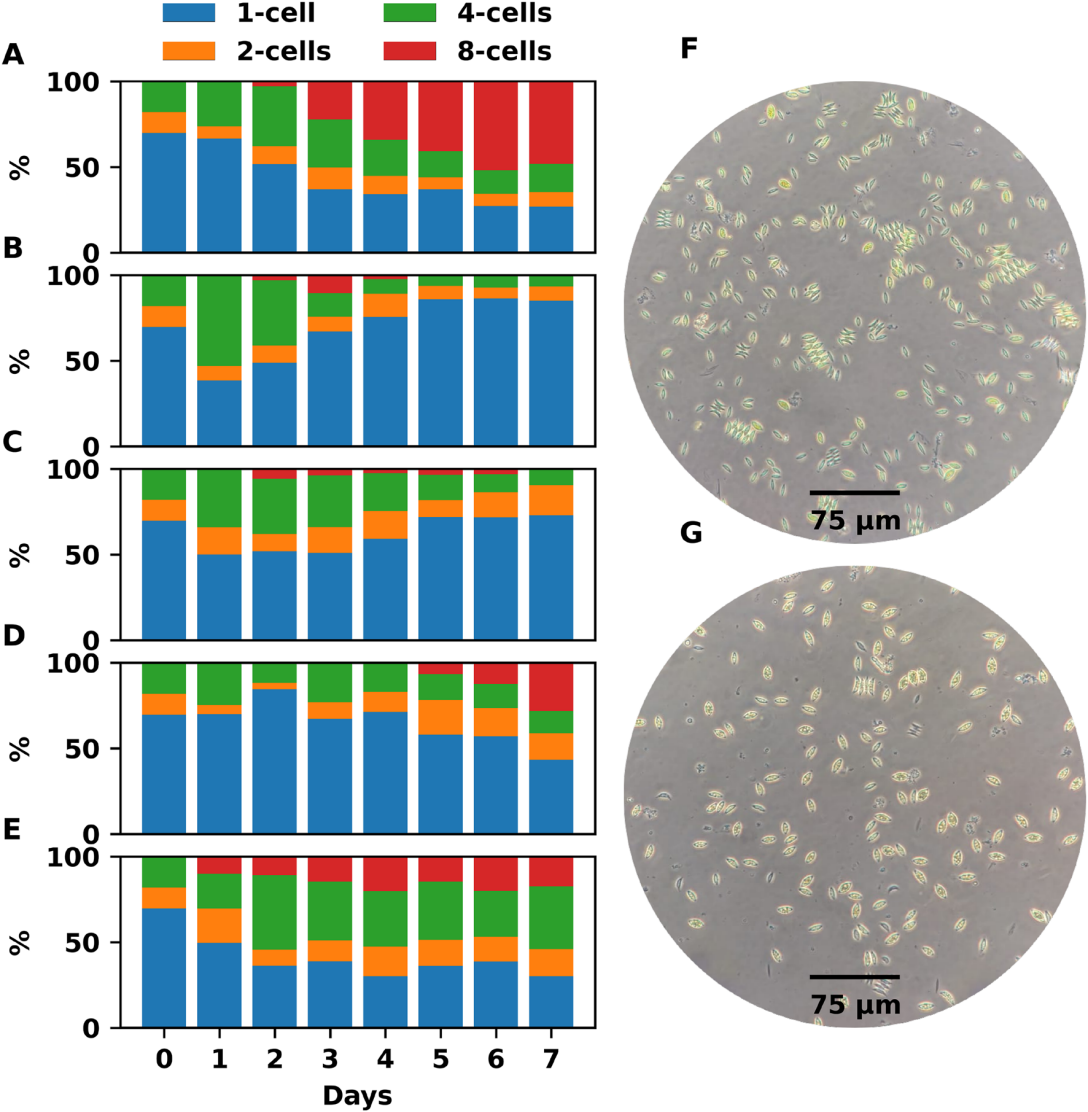
Population structure of the microalgal cultures. The population structure of *S*. *acutus* expressed in percentage, where (**A**–**E**) represent the control, 20, 40, 60, and 80% treatments, respectively. Clear differences between 8-cell and 1-cell cenobium populations for the control and 20% AES treatment can be discerned in figures (**F**) and (**G**), respectively.

The population structure of *S*. *acutus* was also strongly influenced by nutrient availability, which can be differentiated on the last day of cultivation. A greater presence of 8-cell cenobium (48.24%) was observed in the control treatment (Fig. 4A and F). Whereas, cells of 1-cenobium are found in a major percentage (84.95%) in the cultures with 20% of AES (Fig. 4B and G). Cultures with 40% medium similarly presented a high percentage (72.85%) of 1-cell cenobium (Fig. 4C). Meanwhile, cultures with 60% AES (Fig. 4D) also presented a higher percentage of 1-cell cenobium; although, 8-cell cenobium was in a high percentage (28.15%) as well. In cultures with 80% of the medium, 4-cell cenobium dominated, despite the low population growth (Fig. 4E).

An interesting event observed was the different population structures of *S*. *acutus* in the experimental and control treatments. *S*. *acutus* is a microalga that grows forming cenobium of 1, 2, 4, and 8 cells. It has been suggested that the formation of cenobium is a defense mechanism against grazers^78^. Moreover, it has been reported that the formation of larger cenobium is an indicator of optimal growth^79^. This leads to better biomass production. The number of cenobium can also be related to the number of divisions since it is plausible to hypothesize that cells with “n” cenobium will produce “n” generations; therefore, the higher the number of cenobium, the higher the number of daughter cells.

Although the molecular mechanisms of cenobium formation have not been fully understood, we infer that they are mainly influenced by the adaptability of the microalgae to the environment. We observed that 8-cell cenobium was more related to biomass and pigment production than to lipid production. Therefore, we propose that the metabolic pathways that produce the structural machinery that maintains the formation of cenobium (cell linked to cell) are affected by the lack of nitrogen or phosphorus. This requires further study. In addition, quantifying population structure may have relevance to the direction of microalgae production. In this regard, cells with larger cenobium present larger areas and therefore can be more easily centrifuged and/or sedimented, reducing the cost of harvesting.

### Lipid content

**Figure 5:**
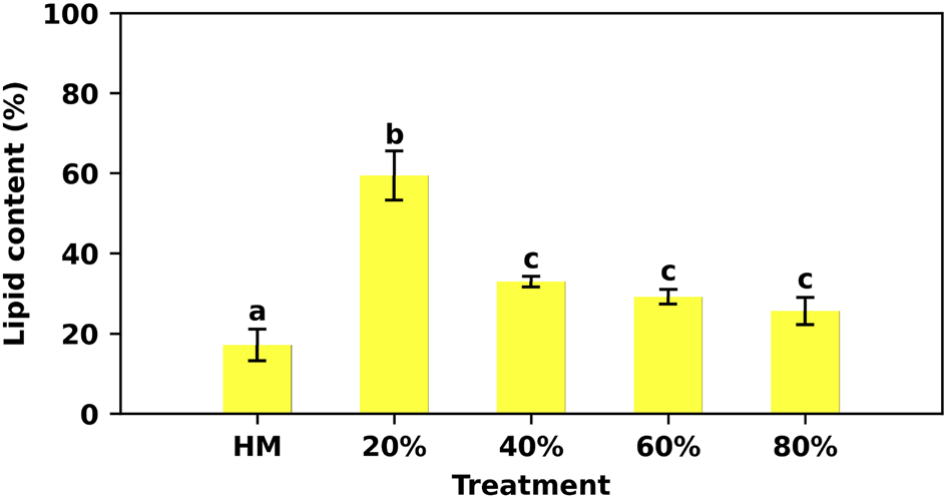
Total lipid content of the microalgal cultures. Total lipid content is shown as a percentage for each of the experiments. The letters above the bars represent the statistical differences between treatments (*p* < 0.05). Cultures with 20 % AES provide high amounts of lipids.

We found that the highest lipid percentage (59.42 ± 6.16) was achieved with 20% AES medium, being in agreement with the lowest level of nutrients supplied. The treatments with 40, 60, and 80% of AES produced lower lipid percentages (33.01 ± 1.37, 29.25 ± 1.83, and 25.66 ± 3.42%, respectively), with no significant statistical difference ($p <$ 0.05). On the other hand, the control treatment presented a total lipid content of 17.19 ± 3.97%, the lowest of all the experimental treatments (Fig. 5).

Lipid production in microalgae can be induced by physiological stress. Reports of *S*. *obliquus* cultures under low levels of nitrogen indicate that it can reach a total of 30.77%^80^. Under this condition, microalgae tend to exhibit structural changes such as increased volume for better nitrogen absorption^81^. We did not observe changes in the morphology of individual cells, but we did observe changes in the population structure and pigmentation of the cultures. Therefore, we can propose that the formation of cenobium and lipid synthesis are inversely related events. Moreover, our findings suggest that the studied strain of *S*. *acutus* posses a higher lipid synthesis capacity, up to 59.42 ± 6.16% of total lipids with 20% AES medium, compared to other *Scenedesmus* species. However, this fact could also be influenced by the characteristics of the experimental medium.

Even though lipid production is mainly affected by N and P, it has been claimed that it is possible to force lipid synthesis by altering other parameters such as light intensity and salinity^82^. Other research indicates that phosphorus deprivation in the medium (less than 2 ppm) causes a decrease in the formation of pyrenoids, the CO_2_-fixing machinery^83^. This drives us to infer that the characteristics of low pigmentation and increased lipid content are gaited in part by this event, strongly demonstrated in the treatment with 20% of AES medium ($\approx$2.18 ppm of P). Further ultrastructural examinations may corroborate this.

Furthermore, microalgae are a promising source of lipids, such as EPA and DHA, which are currently obtained mainly from fish oil^84^. The use of residual material however limits the use of these microalgae lipids for human consumption. Nevertheless, such lipids can be used in the production of biofuels. The quality of the lipids form with AES medium needs to be determined since higher amounts of oleic acid are preferable for biofuel production purposes^85^.

### Parameters and correlations

**Figure 6:**
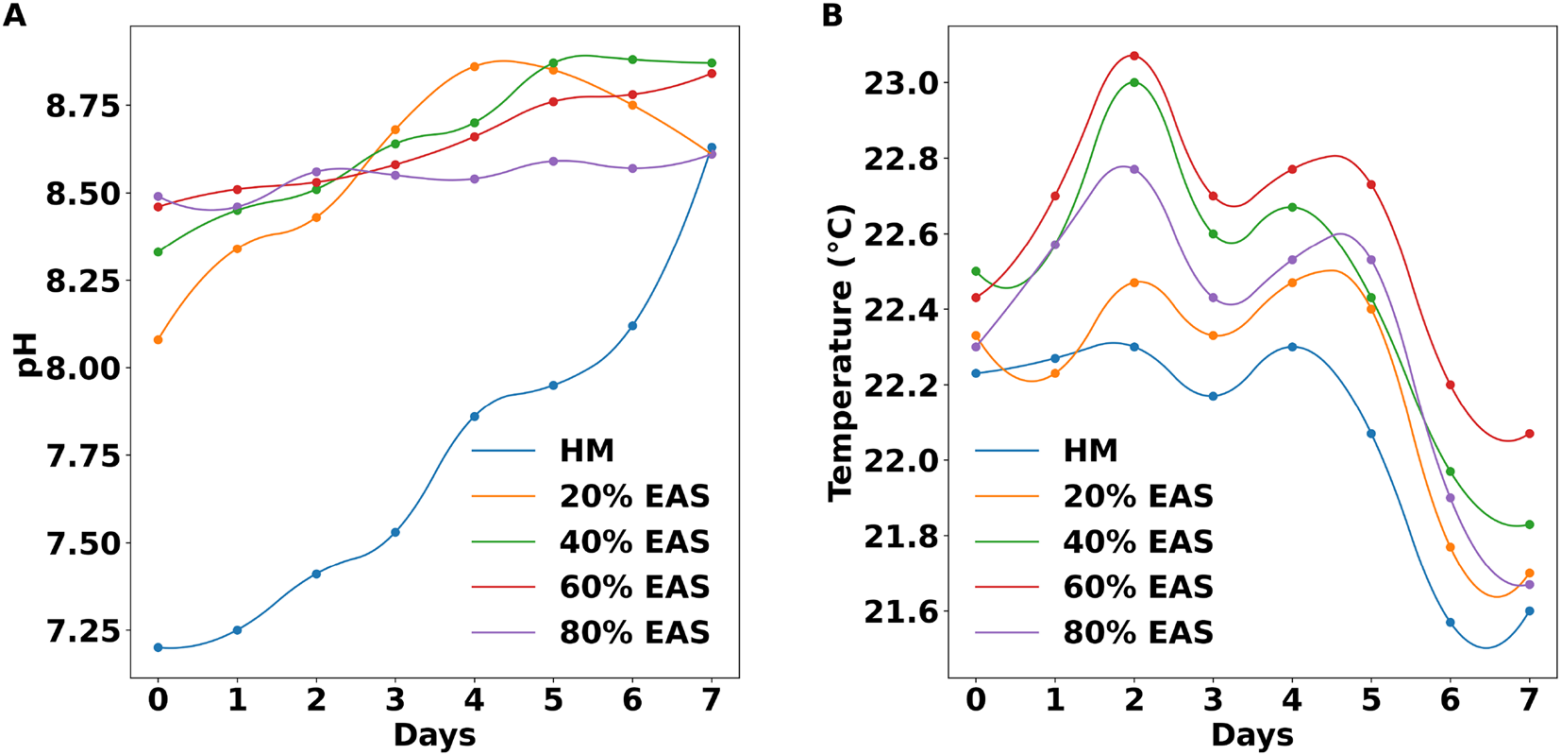
pH and temperature of the microalgal cultures. (**A**) There can be observed marked differences in the initial pH between the control and experimental treatments. (**B**) Temperature fluctuations are slight and within the appropriate range for optimal growth.

The average initial pH in the control cultures was 7.20 units, while in the experimental cultures, the initial pH was > 8.08 units. In all cultures, the pH tended to increase, except in the 20% AES treatment, where the pH decreased moderately after day 4 (Fig. 6A). Meanwhile, temperature maintained a slight fluctuation within the appropriate range, between 21.57 and 23.07 ^∘^C (Fig. 6B).

**Figure 7:**
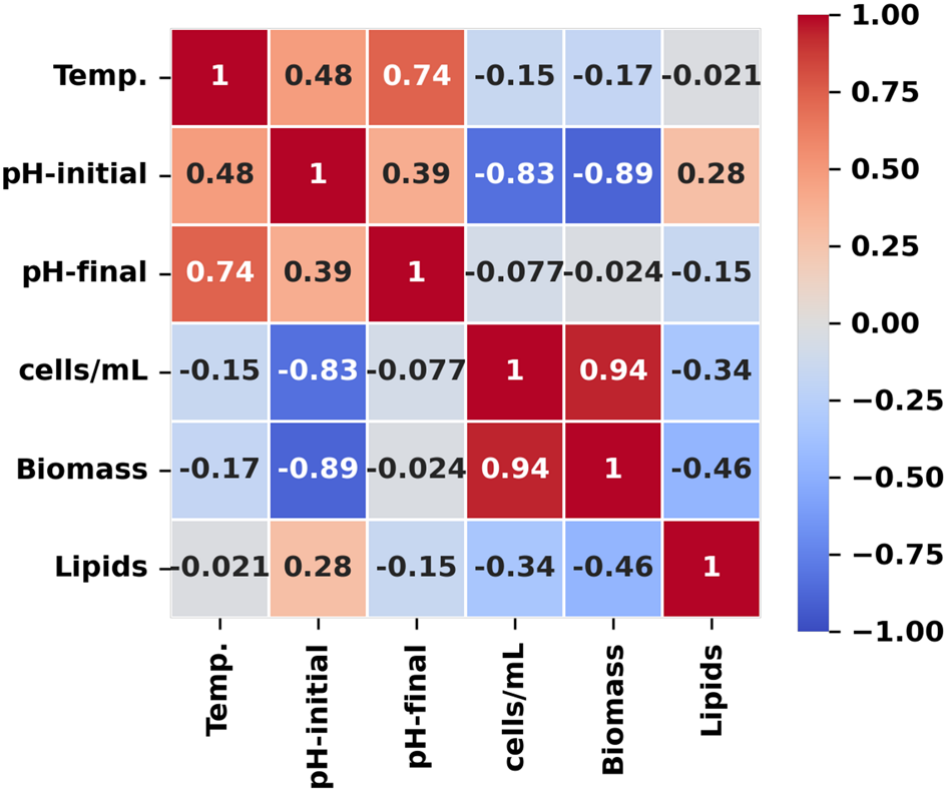
Correlation analysis. The correlations between the main parameters collected indicate that the initial pH of the cultures correlates inversely with growth (cells/mL and biomass). A slight correlation between biomass and lipid content is also observed.

**Table 2:** Statistical analysis of the main growth variables in day 7.

Variables	HM	20%	40%	60%	80%
$\mu$	1.04 ± 0.04^a^	0.90 ± 0.11^b^	0.91 ± 0.02^b^	0.95 ± 0.01^b^	0.74 ± 0.04^c^
*Div*./*day*	1.50 ± 0.06^a^	1.30 ± 0.02^b^	1.31 ± 0.01^b^	1.37 ± 0.01^b^	1.06 ± 0.06^c^
*DT* (days)	0.67 ± 0.03^a^	0.77 ± 0.01^b^	0.76 ± 0.01^b^	0.73 ± 0.01^ab^	0.94 ± 0.05^c^
Biomass (g/L)	1.69 ± 0.14^a^	0.50 ± 0.03^b^	0.64 ± 0.13^b^	0.75 ± 0.07^b^	0.17 ± 0.04^c^

*Different letters indicate significant differences between groups (*p* < 0.05).

In the correlation analysis, we found strong and medium correlations (Fig. 7). The highest correlation found was between biomass (cells/mL) and pH-initial of the culture (-0.89, C.I. = [-0.35;-0.96]). Similarly, cell density (cells/mL) showed a high inverse correlation with initial pH (-0.83, C.I. = [-0.41;-0.95]). Both correlations were significant at a level of *p* < 0.05. An important correlation to note is that given between biomass and lipids (-0.46, C.I. = [0.09;-0.75]), since it denotes a certain level of inverse correlation that agrees with the theory, although not statistically significant was found (Table 3).

**Table 3:** Correlation coefficients and confidence intervals of the main parameters.

Correlations	r^2^	*p*-value	C.I. (95%)	
			2.5%	97.5%
Biomass and pH-initial	− 0.89	1.2 e^-4^*	− 0.35	− 0.96
Cells/mL and pH-initial	− 0.83	1.0 e^-5^*	− 0.41	− 0.95
Biomass and Lipids	− 0.46	8.2 e^-2^	0.09	− 0.75

*Indicates statistically significant difference (*p* < 0.05).

Confidence Intervals (C.I.) have been calculated with 5000 resamples.

Determining correlations between any cultivation parameter and yields is of vital importance to optimize cultures. From our analyses, the highest correlation was found with initial pH and growth (inverse correlation), expressed in cell density and biomass. *Scenedesmus* spp. has an optimal pH range between 7.0 and 8.0^86^; although it tolerates pH ranges up to 9.0^87^. Therefore, the experimental cultures that showed pH values higher than 8.0 affect growth. Furthermore, the bootstrapping analysis indicates that the correlation coefficients can be between -0.35 and -0.96. Based on these results, we can infer that in some cases a not-so-strong correlation could be observed. Thus, we assume that other undetermined factors, such as the effect of toxic elements and/or the absence of some micronutrients (not determined in this study) may be affecting growth.

On the other hand, we have not identified a significant correlation between biomass and lipid quantity, unlike other studies that suggest that biomass is limited by lipid synthesis as a consequence of nitrogen limitation^88^. Hence, the factor inducing lipid synthesis must be more related to the chemical composition of the medium.

## Techno-economic analysis

The techno-economic analysis of the experimental medium compared to other media reveals significant differences between them (Table 4). The conventional mediums display a wide cost range, from 1.17 to 49.62 USD per m^3^, which means high production costs for many industries. In contrast, the HM medium presents a lower cost (2.34 USD per m^3^), being an economical medium as it is elaborated with inputs obtained from conventional agriculture. Meanwhile, the AES medium resulted in a more economical alternative medium than the previously mentioned ones, with a cost of only 0.91 USD per m^3^. Given these results, we consider that scaling up *S*. *acutus* cultures to higher volumes using AES medium will considerably reduce culture and production costs, being necessary to evaluate the economic feasibility under these conditions in future studies. However, it’s crucial to weigh this cost-effectiveness against specific project requirements and performance, as the final decision on the choice of culture medium will depend on the purpose of the project.

Likewise, in circumstances of outdoor cultivation, AES medium results in a green technology that promotes CO_2_ capture through the photosynthetic process of microalgae. It is here where it would be feasible to estimate the greenness index aimed at calculating the large-scale environmental benefits^89^. Finally, it is important to underline that the use of AES medium implies the removal of pollutants from the bay and therefore an improvement of environmental conditions, which has an incalculable intrinsic value.

**Table 4:** Technical-economical analysis of the AES medium compared to other media used in the cultivation of the microalga *S*. *acutus*.

Medium	Cost (USD) per m^3^
Conventional mediums	1.17–49.62^90^
HM medium	2.34*
AES medium	0.91**

*Estimated local market price of the HM-medium components: 206 g urea (CH_4_N_2_O), 19 g KCl, 2.5 g iron, 250 mL HCl (10%), and 25 mL H_3_PO_4_. **Estimated local price based on the cost of dredging, transportation, and boiling derived from this study.

## Conclusion

The chemical characterization of the nutrients of the AES medium represents the first study of the pollutants derived from an organic sludge sampled in El Ferrol Bay. High concentrations of elements, such as Cu, Pb, P, and N, indicate a threatened ecosystem. Thus, the protocol achieved demonstrates that these environmental passives can be extracted by the thermal process, as well as other elements that are used for the production of microalgae. The different concentrations assessed suggest that the use of AES medium would work well from 20 to 60%. The maximum biomass production (0.75 g/L) was obtained with 60% AES; while the maximum lipid production (59.42%) was achieved at 20% AES. In addition, we observed modifications in the population structure of *S*. *acutus* that suggest 8-cell cenobium at higher N and P concentrations. Finally, this work determined that organic nutrients, COD, and TOC can be reduced from the AES medium after 7 days of culture, especially with 20% of AES medium, whichs has important environmental implications.

### Supplementary Information

Supplementary Information.

Supplementary Information.

## Data Availability

All data generated or analyzed during this study are included in this published article and its Supplementary Information files.
