# Supplementary material for: Potential use of sludge from El Ferrol Bay (Chimbote, Peru) for the production of lipids in the culture of Scenedesmus acutus (Meyen, 1829)
Source: Sci Rep. 2024 Mar 23;14:6968. doi: 10.1038/s41598-024-52919-2 (PMC10960819; doi:10.1038/s41598-024-52919-2)
Supplement: Supplementary file 1 [file 41598_2024_52919_MOESM2_ESM.odt]

Supplementary material

A

B

Supplementary Figure 1. A) Scenedesmus acutus strain. B) Inoculum cryopreserved at 4 °C.

B

A

Supplementary Figure 2. A) Sludge sample from Chimbote Bay. B) Final aqueous medium for culture.

Supplementary Table 1.  Heussler-Merino culture medium composition. Each component is prepared with tap water and then used in a concentration of 1 mL/L of culture.

| Compound | Concentration |
| --- | --- |
| Urea (CH₄N₂O) | 206 g/L |
| Phosphoric acid (H3PO4) 100% | 35 mL/L |
| Potassium chloride (KCl) | 19 g/L |
| Iron solution (Fe)* | 2.5 g/L |

*The iron solution is prepared with iron nails diluted in 100 mL of commercial chloridric acid (20%).

Supplementary Table 2. Chemical characterization of the medium by Inductively Coupled Plasma (ICP).

| Total metals | L.C. (mg/L) | AES | Total metals | L.C. (mg/L) | AES |
| --- | --- | --- | --- | --- | --- |
| Ag | 0,002 | <0,002 | Mg | 0,02 | 285,00 |
| Al | 0,02 | 10,33 | Mn | 0,0003 | 0,0902 |
| As | 0,005 | 1,923 | Mo | 0,002 | 0,053 |
| B | 0,003 | 3,179 | Na | 0,06 | >1000 |
| Ba | 0,003 | 0,202 | Ni | 0,0006 | 0,0232 |
| Be | 0,0002 | <0,0002 | P | 0,01 | 10,94 |
| Ca | 0,02 | 138,50 | Pb | 0,002 | 0,080 |
| Cd | 0,0001 | 0,0028 | Sb | 0,003 | <0,003 |
| Ce | 0,009 | 0,012 | Se | 0,005 | 0,013 |
| Co | 0,0006 | 0,0045 | SiO2 | 0,01 | >100 |
| Cr | 0,0003 | 0,0198 | Sn | 0.003 | <0,003 |
| Cu | 0,002 | 0,072 | Sr | 0,0003 | 2,6880 |
| Fe | 0,002 | 11,080 | Ti | 0,0007 | 0,1719 |
| Hg | 0,001 | <0,001 | Tl | 0.002 | <0,002 |
| K | 0,1 | 298,7 | V | 0.001 | 0.066 |
| Li | 0,003 | 0,305 | Zn | 0.002 | 0.139 |

Supplementary Table 3. Growth, biomass and lipid production data.

| Group | µ | Div./day | DT | Biomass | Lipids |
| --- | --- | --- | --- | --- | --- |
| HM R1 | 0.99 | 1.43 | 0.70 | 1.66 | 13.16 |
| HM R2 | 1.07 | 1.54 | 0.65 | 1.84 | 17.31 |
| HM R3 | 1.05 | 1.52 | 0.66 | 1.56 | 21.1 |
| 20 % R1 | 0.92 | 1.33 | 0.75 | 0.47 | 62.94 |
| 20 % R2 | 0.90 | 1.30 | 0.77 | 0.49 | 63.01 |
| 20 % R3 | 0.89 | 1.28 | 0.78 | 0.53 | 52.31 |
| 40 % R1 | 0.91 | 1.32 | 0.76 | 0.55 | 31.53 |
| 40 % R2 | 0.91 | 1.31 | 0.76 | 0.57 | 33.29 |
| 40 % R3 | 0.90 | 1.3 | 0.77 | 0.79 | 34.22 |
| 60 % R1 | 0.95 | 1.37 | 0.73 | 0.74 | 30.68 |
| 60 % R2 | 0.95 | 1.38 | 0.73 | 0.82 | 29.88 |
| 60 % R3 | 0.95 | 1.37 | 0.73 | 0.68 | 27.19 |
| 80 % R1 | 0.78 | 1.13 | 0.89 | 0.12 | 29.95 |
| 80 % R2 | 0.72 | 1.04 | 0.96 | 0.18 | 25.9 |
| 80 % R3 | 0.71 | 1.02 | 0.98 | 0.20 | 22.12 |

* R means repetition.

Supplementary Table 4. Cell density (cells/mL) of S. acutus.

| Group | Day 0 | Day 1 | Day 2 | Day 3 | Day 4 | Day 5 | Day 6 | Day 7 |
| --- | --- | --- | --- | --- | --- | --- | --- | --- |
| HM R1 | 66 | 60 | 86 | 149 | 204 | 555 | 863 | 1100 |
| HM R2 | 66 | 59 | 103 | 124 | 351 | 583 | 859 | 1819 |
| HM R3 | 66 | 48 | 75 | 155 | 473 | 639 | 1176 | 1625 |
| 20 % R1 | 66 | 80 | 106 | 151 | 489 | 445 | 604 | 692 |
| 20 % R2 | 66 | 76 | 71 | 190 | 404 | 457 | 600 | 622 |
| 20 % R3 | 66 | 55 | 85 | 185 | 416 | 473 | 598 | 573 |
| 40 % R1 | 66 | 55 | 88 | 134 | 237 | 375 | 503 | 658 |
| 40 % R2 | 66 | 51 | 88 | 139 | 191 | 375 | 515 | 651 |
| 40 % R3 | 66 | 46 | 98 | 138 | 174 | 408 | 495 | 606 |
| 60 % R1 | 66 | 50 | 43 | 65 | 143 | 313 | 438 | 820 |
| 60 % R2 | 66 | 37 | 70 | 79 | 128 | 264 | 439 | 862 |
| 60 % R3 | 66 | 26 | 69 | 82 | 103 | 249 | 404 | 819 |
| 80 % R1 | 66 | 80 | 49 | 54 | 59 | 146 | 163 | 304 |
| 80 % R2 | 66 | 46 | 58 | 60 | 66 | 97 | 115 | 219 |
| 80 % R3 | 66 | 32 | 40 | 49 | 72 | 85 | 122 | 209 |

* R means repetition.

Supplementary Table 5. Population structure of S. acutus. Cenobiums of 1, 2, 4, and 8 cells were counted.

Supplementary Table 6. Recording of temperature (°C).

| Group | Day 0 | Day 1 | Day 2 | Day 3 | Day 4 | Day 5 | Day 6 | Day 7 |
| --- | --- | --- | --- | --- | --- | --- | --- | --- |
| HM R1 | 22.3 | 22.4 | 22.4 | 22.2 | 22.3 | 22.0 | 21.4 | 21.6 |
| HM R2 | 22.2 | 22.3 | 22.1 | 22.2 | 22.3 | 22.0 | 21.6 | 21.5 |
| HM R3 | 22.2 | 22.1 | 22.4 | 22.1 | 22.3 | 22.2 | 21.7 | 21.7 |
| 20 % R1 | 22.2 | 22.1 | 22.4 | 22.2 | 22.5 | 22.4 | 21.6 | 21.6 |
| 20 % R2 | 22.4 | 22.2 | 22.6 | 22.4 | 22.4 | 22.4 | 21.9 | 21.7 |
| 20 % R3 | 22.4 | 22.4 | 22.4 | 22.4 | 22.5 | 22.4 | 21.8 | 21.8 |
| 40 % R1 | 22.5 | 22.8 | 23.0 | 22.7 | 22.6 | 22.4 | 21.8 | 21.8 |
| 40 % R2 | 22.5 | 22.4 | 23.0 | 22.5 | 22.6 | 22.4 | 22.0 | 21.8 |
| 40 % R3 | 22.5 | 22.5 | 23.0 | 22.6 | 22.8 | 22.5 | 22.1 | 21.9 |
| 60 % R1 | 22.5 | 22.5 | 23.1 | 22.6 | 22.7 | 22.6 | 21.9 | 21.8 |
| 60 % R2 | 22.5 | 22.6 | 23.0 | 22.7 | 22.7 | 22.8 | 22.2 | 22.1 |
| 60 % R3 | 22.3 | 23.0 | 23.1 | 22.8 | 22.9 | 22.8 | 22.5 | 22.3 |
| 80 % R1 | 22.3 | 22.5 | 23.0 | 22.6 | 22.8 | 22.8 | 21.9 | 21.5 |
| 80 % R2 | 22.3 | 22.5 | 22.7 | 22.4 | 22.5 | 22.5 | 22.0 | 21.7 |
| 80 % R3 | 22.3 | 22.7 | 22.6 | 22.3 | 22.3 | 22.3 | 21.8 | 21.8 |

* R means repetition.

Supplementary Table 7. Recording of pH.

| Group | Day 0 | Day 1 | Day 2 | Day 3 | Day 4 | Day 5 | Day 6 | Day 7 |
| --- | --- | --- | --- | --- | --- | --- | --- | --- |
| HM R1 | 7.21 | 7.22 | 7.53 | 7.54 | 7.89 | 7.90 | 7.96 | 8.65 |
| HM R2 | 7.20 | 7.3 | 7.41 | 7.57 | 7.84 | 7.81 | 8.18 | 8.63 |
| HM R3 | 7.20 | 7.23 | 7.29 | 7.49 | 7.85 | 8.15 | 8.23 | 8.60 |
| 20 % R1 | 8.15 | 8.26 | 8.35 | 8.66 | 8.81 | 8.81 | 8.65 | 8.53 |
| 20 % R2 | 8.10 | 8.38 | 8.46 | 8.68 | 8.82 | 8.92 | 8.84 | 8.67 |
| 20 % R3 | 8.00 | 8.37 | 8.48 | 8.69 | 8.95 | 8.82 | 8.76 | 8.64 |
| 40 % R1 | 8.31 | 8.44 | 8.50 | 8.65 | 8.64 | 8.85 | 8.83 | 8.85 |
| 40 % R2 | 8.37 | 8.42 | 8.51 | 8.61 | 8.69 | 8.81 | 8.84 | 8.85 |
| 40 % R3 | 8.32 | 8.48 | 8.52 | 8.65 | 8.76 | 8.96 | 8.96 | 8.92 |
| 60 % R1 | 8.41 | 8.49 | 8.50 | 8.57 | 8.68 | 8.72 | 8.61 | 8.84 |
| 60 % R2 | 8.49 | 8.52 | 8.54 | 8.58 | 8.72 | 8.79 | 8.88 | 8.81 |
| 60 % R3 | 8.48 | 8.51 | 8.55 | 8.58 | 8.59 | 8.77 | 8.85 | 8.87 |
| 80 % R1 | 8.48 | 8.43 | 8.54 | 8.53 | 8.54 | 8.61 | 8.56 | 8.49 |
| 80 % R2 | 8.49 | 8.46 | 8.56 | 8.55 | 8.52 | 8.59 | 8.58 | 8.62 |
| 80 % R3 | 8.49 | 8.48 | 8.57 | 8.56 | 8.55 | 8.56 | 8.58 | 8.73 |

* R means repetition.
